# Characterization of N-terminally mutated cardiac Na^+^ channels associated with long QT syndrome 3 and Brugada syndrome

**DOI:** 10.3389/fphys.2013.00153

**Published:** 2013-06-26

**Authors:** Christian Gütter, Klaus Benndorf, Thomas Zimmer

**Affiliations:** Institute of Physiology II, University Hospital Jena, Friedrich Schiller University JenaJena, Germany

**Keywords:** cardiac sodium channel, cardiac arrhythmia, *SCN5A* channelopathies, electrophysiology, Long QT syndrome, Brugada syndrome, N-terminus

## Abstract

Mutations in *SCN5A*, the gene encoding the cardiac voltage-gated Na^+^ channel hNa_v_1.5, can result in life-threatening arrhythmias including long QT syndrome 3 (LQT3) and Brugada syndrome (BrS). Numerous mutant hNa_v_1.5 channels have been characterized upon heterologous expression and patch-clamp recordings during the last decade. These studies revealed functionally important regions in hNa_v_1.5 and provided insight into gain-of-function or loss-of-function channel defects underlying LQT3 or BrS, respectively. The N-terminal region of hNa_v_1.5, however, has not yet been investigated in detail, although several mutations were reported in the literature. In the present study we investigated three mutant channels, previously associated with LQT3 (G9V, R18W, V125L), and six mutant channels, associated with BrS (R18Q, R27H, G35S, V95I, R104Q, K126E). We applied both the two-microelectrode voltage clamp technique, using cRNA-injected *Xenopus* oocytes, and the whole-cell patch clamp technique using transfected HEK293 cells. Surprisingly, four out of the nine mutations did not affect channel properties. Gain-of-function, as typically observed in LQT3 mutant channels, was observed only in R18W and V125L, whereas loss-of-function, frequently found in BrS mutants, was found only in R27H, R104Q, and K126E. Our results indicate that the hNa_v_1.5 N-terminus plays an important role for channel kinetics and stability. At the same time, we suggest that additional mechanisms, as e.g., disturbed interactions of the Na^+^ channel N-terminus with other proteins, contribute to severe clinical phenotypes.

## Introduction

Voltage-gated sodium (Na^+^) channels are responsible for the rapid upstroke of the action potential in electrically excitable cells. The tetrodotoxin (TTX) resistant isoform hNa_v_1.5, encoded by the *SCN5A* gene, is the predominant isoform in the human heart (Gellens et al., 1992; Blechschmidt et al., 2008; Zimmer, 2010; Rook et al., 2012; Savio-Galimberti et al., 2012). A broad spectrum of mutations in *SCN5A* were related to a variety of inherited cardiac diseases, such as long QT syndrome type 3 (LQT3), Brugada syndrome (BrS), cardiac conduction disease (CCD), or sick sinus syndrome (SSS) (Zimmer and Surber, 2008; Gui et al., 2010). Heterologous expression of respective mutant hNa_v_1.5 channels revealed important insight into the mechanisms underlying these cardiac diseases. In LQT3, mutant channels are characterized by gain-of-function features, like faster recovery from the inactivated state (Chandra et al., 1998; Clancy et al., 2003), inactivation defects (Bennett et al., 1995; Chandra et al., 1998), or dispersed reopenings from the inactivated state (Dumaine et al., 1996). Such defects are believed to result in an action potential widening and consequently, in the observed QT prolongation. In BrS or CCD, mutant channels often show loss-of-function features, like reduced channel availability at the resting membrane potential (Rivolta et al., 2001), a positive shift of steady-state activation (Vatta et al., 2002a; Potet et al., 2003), impaired trafficking to the plasma membrane (Baroudi et al., 2001; Valdivia et al., 2004), or the inability to conduct Na^+^ (Kyndt et al., 2001; Zhang et al., 2008). Such defects can explain cardiac conduction abnormalities and the observed ST segment elevation (Alings and Wilde, 1999; Yan and Antzelevitch, 1999). These genotype-phenotype associations in *SCN5A* channelopathies are not only important for clinicians regarding the management of genotype-positive patients and symptom-free family members. Functional data on mutant channels also extended our knowledge about important structural elements in the cardiac Na^+^ channel, like the DIII-DIV linker as the inactivation gate (Bennett et al., 1995) or position 1053 for ankyrin-G binding (Mohler et al., 2004).

Currently, however, we are faced with a growing number of mutations that are not yet characterized by electrophysiological measurements. The lack of functional data makes it difficult for clinicians to interpret the results of genetic testing, because a rare deviation from the published *SCN5A* sequence could be malign or even benign. For example, only 20% of the BrS patients are *SCN5A*-positive cases, and the majority of BrS-causative mutations or factors still remain obscure (Kapplinger et al., 2010). Consequently, the same yet unknown factors could be also crucial for the manifestation of the disease in *SCN5A*-positive BrS patients, in particular when mutant channels were either not characterized or electrophysiologically indiscernible from wild-type hNa_v_1.5.

**Figure 1:**
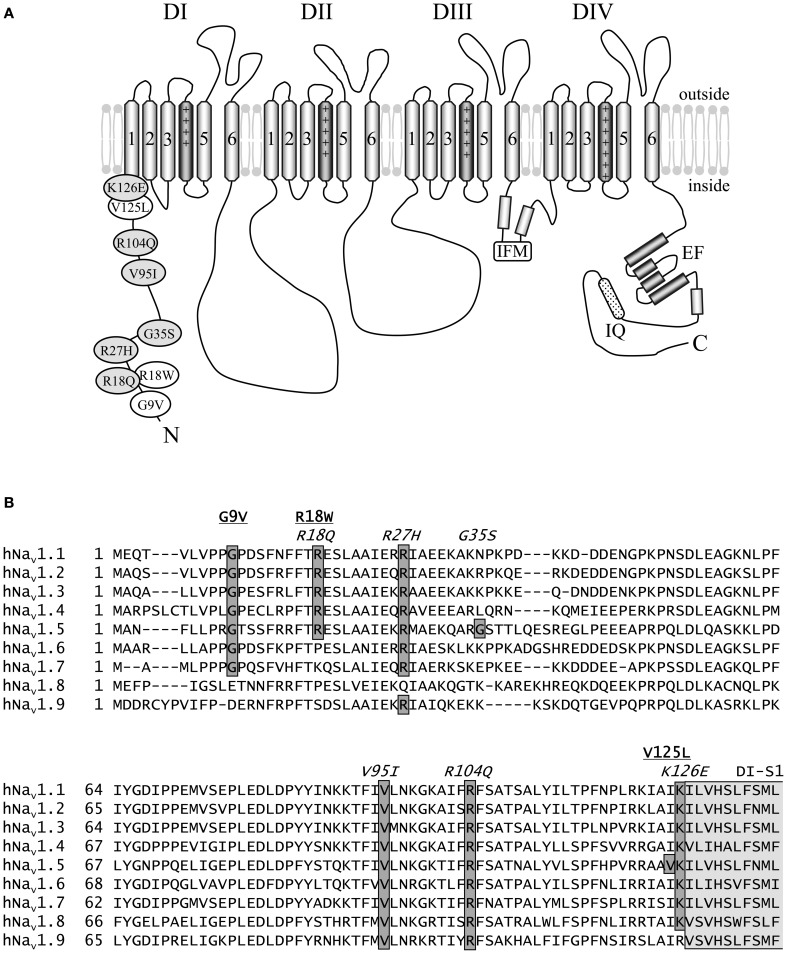
**Schematic representation of hNa_v_1.5 and the N-terminal mutations investigated in this study. (A)** Proposed hNa_v_1.5 topology. Affected residues are indicated in white (LQT3) and light grey (BrS). The schematic structure also highlights some important structural features (DI to DIV—domain I to IV, IFM—residues isoleucine, phenylalanine, and methionine of the inactivation gate, IQ—calmodulin binding motif, EF—Ca^++^ binding EF hand domain). **(B)** Alignment of the N-terminal sequences of human Nav1.1—Nav1.9. Mutations associated with LQT3 or BrS are underlined or indicated in italics, respectively. Most of the eight affected residues are conserved among the Na_v_1 subfamily. Residues at position 35 are variable, and position 125 is occupied by isoleucine in all other human Na^+^ channels. All eight residues affected by a *SCN5A* mutation are identical in Na_v_1.5 of human, rat, mouse, and dog (not shown). The N-terminal region of the first putative membrane spanning segment DI-S1 is indicated in light grey. References: G9V (Millat et al., 2006), R18W (Tester et al., 2005), R18Q (Kapplinger et al., 2010), R27H (Priori et al., 2002), G35S (Levy-Nissenbaum et al., 2001), V95I (Liang et al., 2006), R104Q (Levy-Nissenbaum et al., 2001), V125L (Tester et al., 2005), K126E (Vatta et al., 2002a).

The present study focuses on nine arrhythmia-causing missense mutations localized to the N-terminus of hNa_v_1.5 that have not yet been characterized by electrophysiological techniques (Figure 1). We selected all three published LQT3 mutations and six out of seventeen BrS mutations reported in the online database of Drs. Priori and Napolitano (http://www.fsm.it/cardmoc/). The aim of this project was to establish respective genotype-phenotype correlations and to get more insight into the role of the intracellularly exposed N-terminus for channel gating. This region has not yet been investigated in detail in terms of structure-function relationships. Therefore, it was challenging for us to search for possible inactivation defects in the LQT3 mutant channels, and for loss-of-function features caused by the BrS missense mutations. All mutant channels were expressed in both HEK293 cells and *Xenopus* oocytes. We performed electrophysiological measurements in both heterologous hosts to identify or exclude cell-specific effects.

## Material and methods

### Recombinant DNA procedures

Generation of the expression plasmid pTSV40G-hNa_v_1.5, coding for wild-type hNa_v_1.5, was described previously (Walzik et al., 2011). The original hH1 cDNA (accession number M77235) was kindly provided by Dr. A. L. George (Gellens et al., 1992). Mutations at amino acid positions 9, 18, 27, 35, 95, 104, 125, and 126 were introduced using the recombinant PCR technique and the following internal primer pairs:

5′-TTACCTCGGGTCACCAGCAGCTTCCGCAGG-3′ and
5′-GGAAGCTGCTGGTGACCCGAGGTAATAGGAAGTTTG-3′ to obtain G9V,
5′-GCAGGTTCACACTGGGAGTCCCTGGCAGCCATC-3′ and
5′-CCAGGGACTCCCATGTGAACCTGCGGAAGCTG-3′ to obtain R18W,
5′-GCAGGTTCACACAGGAGTCCCTGGCAGCCATC-3′ and
5′-CCAGGGACTCCTGTGTGAACCTGCGGAAGCTG-3′ to obtain R18Q,
5′-CATCGAGAAGCACATGGCGGAGAAGCAAGCCC-3′ and
5′-CTTCTCCGCCATGTGCTTCTCGATGGCTGCCAGG-3′ to obtain R27H,
5′-AAGCAAGCCCGCAGCTCAACCACCTTGCAGGAG-3′ and
5′-GGTGGTTGAGCTGCGGGCTTGCTTCTCCGCCATG-3′ to obtain G35S,
5′-AAGACTTTCATCATACTGAATAAAGGCAAGACCA-3′ and
5′-CCTTTATTCAGTATGATGAAAGTCTTTTGGGTG-3′ to obtain V95I,
5′-ACCATCTTCCAGTTCAGTGCCACCAACGCCTT-3′ and
5′-TGGCACTGAACTGGAAGATGGTCTTGCCTTTA-3′ to obtain R104Q,
5′-AGAGCGGCTTTGAAGATTCTGGTTCACTCG-3′ and
5′-GAACCAGAATCTTCAAAGCCGCTCTCCGGATGGGGTGG-3′ to obtain V125L, and
5′-CGGCTGTGGAGATTCTGGTTCACTCGCTCTT-3′ and
5′-AGTGAACCAGAATCTCCACAGCCGCTCTCCGGATGGGGT-3′

to obtain K126E. The recombinant PCR products were inserted as *Age*I/*Hind*III fragments into the corresponding sites of pTSV40G-hNa_v_1.5. A thermostable DNA polymerase with proofreading activity was used for all PCR reactions (Pfu DNA polymerase, Promega, Madison, USA). The correctness of PCR-derived sequences was confirmed by DNA sequencing. Construction of the control plasmid encoding ΔKPQ channels was previously described (Surber et al., 2008). All channel variants were placed under the control of the SV40 promoter in expression plasmid pTSV40G, a derivative of pTracerSV40 (Invitrogen) (Camacho et al., 2006). Vector pTSV40G contains the T7 promoter and, in a separate expression cassette, the coding region of the enhanced green fluorescent protein (EGFP; Clontech) to allow for cRNA preparation and for selection of transfected HEK293 cells, respectively.

### Heterologous expression in HEK293 cells

Heterologous expression in mammalian cells was done as previously described (Zimmer et al., 2002b). Human embryonic kidney cells (HEK293 cell line, ATCC number CRL-1573) were cultured in Dulbecco's Modified Eagle Medium (DMEM; GibcoBRL), supplemented with 2 mM glutamine, 10% fetal bovine serum, 100 μg/ml streptomycin, 100 U/ml penicillin, and 0.25 μg/ml amphotericin B. HEK293 cells were transfected by a standard calcium phosphate precipitation method using 1.0 μg plasmid DNA per transfection dish (60 mm diameter). After an incubation time of 24 h, the transfection mixture was removed. Cells were seeded onto poly-L-lysine coated glass cover slips and cultured in fresh growth medium. Na^+^ currents were investigated 24–48 h after transfection.

### Patch-clamp measurements

Electrophysiological recordings were performed, as previously described (Surber et al., 2008; Walzik et al., 2011). We used an inverted microscope (Axiovert 100, Carl Zeiss Jena GmbH, Germany) and an Axopatch 200B amplifier (Axon-Instruments, Foster City, USA). All measurements were carried out at room temperature (19–22°C). The bath solution contained (mM): 140.0 NaCl, 1.8 CaCl_2_, 1.0 MgCl_2_, 10.0 glucose, 10.0 HEPES, pH 7.4 (CsOH). The pipette solution contained (mM): 10.0 NaCl, 130.0 CsCl, 10.0 EGTA, 10.0 HEPES, pH 7.3 (CsOH). Currents were elicited by test potentials from −80 mV to 40 mV in 5 or 10 mV increments at a pulsing frequency of 1.0 Hz (holding potential −120 mV). Cells that produced a peak current amplitude >6 nA were excluded from data analysis. Steady-state activation (m_∞_) was evaluated by fitting the Boltzmann equation m_∞_ = {1 + exp[−(*V* − *V*_*m*_)/*s*]}^−1^ to the normalized conductance as function of voltage. Steady-state inactivation (h_∞_) was determined with a double-pulse protocol consisting of 500 ms prepulses to voltages between −140 and −30 mV followed by a constant test pulse of 10 ms duration to −20 mV at a pulsing frequency of 0.5 Hz. The amplitude of peak *I*_Na_ during the test pulse was normalized to the maximum peak current and plotted as function of the prepulse potential. Data were fitted to the Boltzmann equation h_∞_ = {1 + exp[(*V* − *V*_*h*_)/*s*]}^−1^. V is the test potential, *V*_*m*_ and *V*_*h*_ are the mid-activation and mid-inactivation potentials, respectively, and s the slope factor in mV. Glass pipettes were pulled from borosilicate glass. Glass tips were heat polished by microforge MF 830 (Narishige, Japan). The pipette resistance was between 1.4 and 2.6 MΩ. Series resistance compensation was adjusted so that any oscillations were avoided leaving at most 25% of the series resistance uncompensated. Currents were on-line filtered with a cut-off frequency of 10 kHz (4-pole Bessel). Recording and analysis of the data was performed on a personal computer with the ISO3 software (MFK, Niedernhausen, Germany). The sampling rate was 50 kHz. Student's *t*-test was used to test for statistical significance. Statistical significance was assumed for *P* < 0.05.

### Expression in *xenopus laevis* OOCYTES

Preparation of *Xenopus laevis* oocytes, *in vitro* transcription, and cRNA injection was done as previously described (Zimmer et al., 2002a). Fluorescence intensities of the cRNA bands were measured using the gel documentation system from Herolab (Wiesloch, Germany). Concentrations of the cRNA variants were adjusted to ~ 0.01 μg/μl, before injecting about 50–80 nl cRNA per oocyte. After 3 days incubation at 18°C in Barth medium, the peak current amplitude of the whole-cell Na^+^ current was between 0.5 and 8.0 μA, depending on the quality of the oocyte batch. Cells producing currents larger than 5 μA were not selected for data evaluation. Measurements were performed in at least four different batches of oocytes. For the measurements of persistent Na^+^ currents we injected undiluted cRNA preparations (~0.2 μg/μl) in order to increase this small current fraction. This resulted in transient Na^+^ currents >8 μA in 96 mM external Na^+^.

### Two-microelectrode voltage-clamp technique

Whole-cell Na^+^ currents were recorded with the two-microelectrode voltage-clamp technique, similarly as previously described (Zimmer et al., 2002a). For all recordings we used the amplifier TEC-05-S (npi electronic GmbH, Tamm, Germany). For the determination of peak current amplitudes, steady-state activation, steady-state inactivation and recovery from inactivation, the following bath solution was used (in mM): 96 NaCl, 2 KCl, 1.8 CaCl_2_, 1 MgCl_2_, 10 HEPES/KOH, pH 7.4. The persistent current fraction was determined as previously described (Surber et al., 2008): First, we measured the inward current at the end of a 200 ms test pulse that could be blocked by 10 μM TTX in 96 mM external Na^+^ (*I*_persistent_). Then, we reduced the extracellular Na^+^ concentration to 20 mM in order to insure adequate voltage control also for the first few milliseconds of the test pulse and determined the peak current amplitude in the same oocyte (*I*_transient_). The following bath solution was used (in mM): 20 NaCl, 78 KCl, 1.8 CaCl_2_, 1 MgCl_2_, 10 HEPES/KOH, pH 7.4. Currents were elicited by 200 ms test potentials from −80 to 40 mV in 5 mV or 10 mV increments (holding potential −120 mV, pulsing frequency 1.0 Hz).

## Results

### Properties of LQT3 mutant channels (G9V, R18W, V125L)

**Figure 2:**
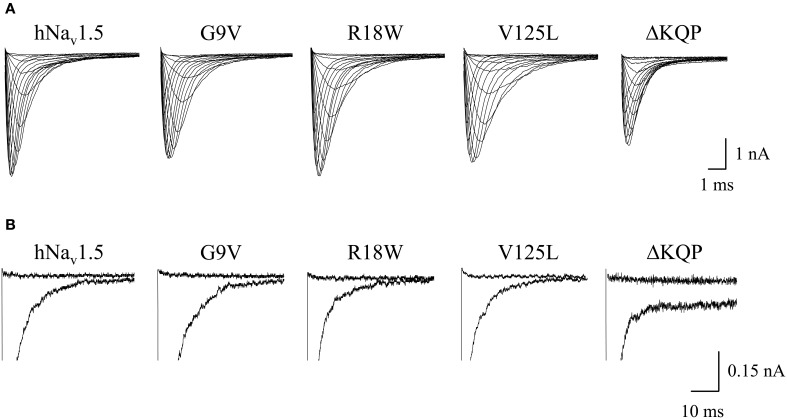
**Whole-cell Na^+^ currents upon expression in HEK293 of hNa_v_1.5 mutant channels associated with LQT3. (A)** Current families. Currents were elicited by test potentials from −80 mV to various test pulses in 5 or 10 mV increments at a pulsing frequency of 1.0 Hz. **(B)** Persistent currents at −20 mV. The non-inactivating current fraction was similarly small in both wild-type and mutant hNa_v_1.5 channels. For individual values see Table 1. Mutant ΔKQP channels were used as a positive control.

**Figure 3:**
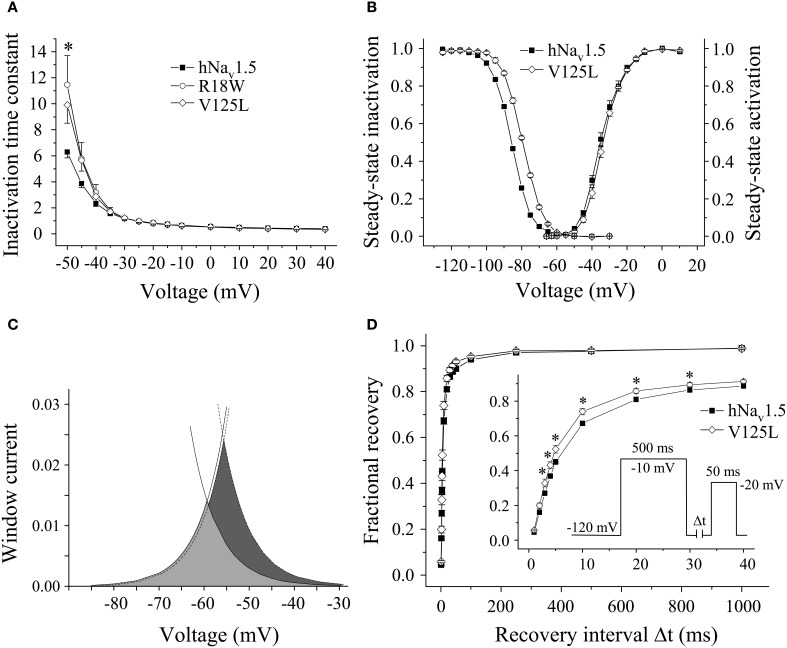
**Electrophysiological properties in HEK293 cells of mutant hNa_v_1.5 channels associated with LQT3. (A)** Inactivation time constants τ_h_ (ms) as function of voltage. At −50 mV both R18W and V125L channels inactivated more slowly compared to hNa_v_1.5 (^*^indicates *p* < 0.05). G9V channel inactivation was indistinguishable from hNa_v_1.5 (data not shown). **(B)** Steady-state activation and inactivation in V125L channels. The figure shows the mean of 4 representative measurements for each. **(C)** Window current in hNa_v_1.5 (grey area, solid lines) and V125L (dark grey area, dotted lines). **(D)** Recovery from inactivation was accelerated in V125L (^*^ indicates *p* < 0.05 vs. hNa_v_1.5). For individual values see Table 2.

All three mutant channels associated with LQT3 generated whole-cell currents comparable to those observed for hNa_v_1.5 (Table 1, Figure 2A). No significant differences were observed in the peak current density when expressing G9V, R18W and V125L in HEK293 cells or in *Xenopus* oocytes. Also, the persistent current fraction was not increased in both expression systems (Table 1, Figure 2B), which is in contrast to mutant ΔKPQ channels that were included as a positive control. When analyzing channel inactivation by fitting the Na^+^ current decay using a mono-exponential function, we observed a slower inactivation at more negative test potentials in R18W and V125L (Figure 3A). Respective inactivation time constants τ_h_ were significantly increased at −50 mV (R18W: τ_h_ = 11.5 ± 2.2, *n* = 12; V125L: τ_h_ = 9.9 ± 1.4, *n* = 14; hNa_v_1.5: τ_h_ = 6.3 ± 0.4, *n* = 55).

**Table 1:** **Peak current densities and persistent currents in HEK293 and *Xenopus laevis* oocytes**.

**Channel**	**HEK239 cells**				***Xenopus laevis* oocytes**				
	**Peak current density**		***I*_persistent_/*I*_transient_**		**Normalized peak current**		***I*_persistent_/*I*_transient_^a^**		
	**at −25 mV (pA/pF)**	***n***	**at −20 mV (%)**	***n***	**at −20 mV**	***n***	**at −30 mV (%)**	**at −10 mV (%)**	***n***
**CONTROL**									
hNa_v_1.5	193 ± 12	90	0.22 ± 0.09	5	1.00 ± 0.04	92	0.83 ± 0.10	1.27 ± 0.23	18
**LQT3**									
G9V	200 ± 33	11	0.25 ± 0.15	4	1.29 ± 0.23	26	1.45 ± 0.30	1.88 ± 0.29	12
R18W	260 ± 36	14	0.19 ± 0.07	4	0.84 ± 0.07	27	1.52 ± 0.70	1.20 ± 0.40	4
V125L	161 ± 28	21	0.08 ± 0.05	4	1.08 ± 0.16	36	1.28 ± 0.36	1.10 ± 0.38	6
**BrS**									
R18Q	175 ± 21	19	n.d.	−	1.26 ± 0.18	23	n.d.	n.d.	−
R27H	159 ± 20	18	n.d.	−	1.51 ± 0.36	19	n.d.	n.d.	−
G35S	224 ± 35	11	n.d.	−	1.09 ± 0.12	20	n.d.	n.d.	−
V95I	205 ± 15	20	n.d.	−	0.88 ± 0.15	23	n.d.	n.d.	−
R104Q	No current	10	n.d.	−	0.29 ± 0.02^*^	45	n.d.	n.d.	−
K126E	172 ± 20	24	n.d.	−	1.13 ± 0.09	36	n.d.	n.d.	−

a *I_persistent_/I_transient_ ratios in ΔKPQ channels: 10.4 ± 2.1 % at −30 mV, 12.8 ± 2.9 % at −10 mV (n = 8)*.

* *indicates p < 0.05 vs. hNa_v_1.5*.

Steady-state activation remained unchanged in all LQT3 mutant channels. Mid-activation potentials *V*_*m*_ were not significantly different from hNa_v_1.5 values in HEK293 cells and *Xenopus* oocytes (Tables 2, 3). Similarly, no differences were observed in channel availability, except for V125L in HEK293 cells (Table 2, Figure 3B). The respective mid-inactivation potential *V*_*h*_ was shifted by 3.1 mV into depolarized direction, when compared to wild-type channels. As a consequence, the window current resulting from the small overlap of the steady-state activation and steady-state inactivation curves should be increased in V125L (Figure 3C). Interestingly, this effect was not seen in the oocyte system: Mid-inactivation potentials were neither altered in V125L nor in the other two LQT3 mutant channels G9V and R18W (Table 3). When analyzing the recovery from inactivation in HEK293 cells using a double pulse protocol we noticed an accelerated recovery in V125L (Figure 3D). The fast time constant τ_*f*_ was significantly smaller and the corresponding amplitude *A*_*f*_ was increased (Table 2). This effect of the mutation at position 125 on recovery from inactivation was not observed in the oocyte system: Recovery time constants and the corresponding amplitudes were similar in all three LQT3 mutant channels, when compared to hNa_v_1.5 data (Table 3).

**Table 2:** **Electrophysiological properties of mutant hNa_v_1.5 channels in HEK293 cells**.

**Channel**	**Steady-state activation**			**Steady-state inactivation**			**Recovery from inactivation**				
	***s* (mV)**	***V*_*m*_ (mV)**	***n***	***s* (mV)**	***V*_*h*_ (mV)**	***n***	**τ_*f*_ (ms)**	***A*_*f*_**	**τ_*s*_ (ms)**	***A*_*s*_**	***n***
**CONTROL**											
hNa_v_1.5	5.7 ± 0.1	−35.1 ± 0.4	88	5.9 ± 0.1	−84.3 ± 0.6	68	5.8 ± 0.3	0.84 ± 0.01	110 ± 17	0.16 ± 0.04	40
**LQT3**											
G9V	5.7 ± 0.4	−33.8 ± 1.3	11	6.0 ± 0.1	−84.2 ± 2.0	8	5.4 ± 0.7	0.81 ± 0.04	60.0 ± 9	0.19 ± 0.03	6
R18W	5.6 ± 0.3	−35.3 ± 2.2	13	5.6 ± 0.2	−83.0 ± 2.4	9	4.8 ± 0.5	0.83 ± 0.05	70.0 ± 24	0.17 ± 0.04	5
V125L	5.6 ± 0.2	−34.4 ± 1.0	21	5.6 ± 0.1	−81.2 ± 0.9^*^	20	4.8 ± 0.3^*^	0.88 ± 0.01^*^	128 ± 22	0.12 ± 0.01^*^	18
**BrS**											
R18Q	5.7 ± 0.2	−34.6 ± 1.1	18	5.7 ± 0.2	−82.1 ± 1.0	16	5.0 ± 0.5	0.82 ± 0.04	52.0 ± 6	0.18 ± 0.03	10
R27H	6.8 ± 0.2^*^	−31.1 ± 1.0^*^	18	5.8 ± 0.2	−84.7 ± 1.2	14	6.4 ± 0.6	0.77 ± 0.03^*^	111 ± 26	0.23 ± 0.03^*^	12
G35S	5.4 ± 0.4	−36.8 ± 1.5	10	5.4 ± 0.1	−86.1 ± 1.3	9	4.7 ± 0.4	0.85 ± 0.01	91.0 ± 23	0.15 ± 0.01	7
V95I	6.0 ± 0.2	−33.9 ± 0.9	20	5.5 ± 0.1	−83.3 ± 1.3	15	5.7 ± 0.6	0.83 ± 0.02	60.0 ± 11	0.17 ± 0.02	9
K126E	5.9 ± 0.3	−32.3 ± 1.1^*^	21	5.9 ± 0.1	−80.9 ± 0.9^*^	21	5.2 ± 0.5	0.86 ± 0.02	91.0 ± 23	0.14 ± 0.02	13

* * indicates p < 0.05 vs. hNa_v_1.5*.

**Table 3:** **Electrophysiological properties of mutant hNa_v_1.5 channels in *Xenopus laevis* oocytes**.

**Channel**	**Steady-state activation**			**Steady-state inactivation**			**Recovery from inactivation**				
	***s* (mV)**	***V*_*m*_ (mV)**	***n***	***s* (mV)**	***V*_*h*_ (mV)**	***n***	**τ_*f*_ (ms)**	***A*_*f*_**	**τ_*s*_ (ms)**	***A*_*s*_**	***n***
**CONTROL**											
hNa_v_1.5	3.6 ± 0.1	−33.5 ± 0.4	82	5.4 ± 0.1	−71.2 ± 0.7	28	3.8 ± 0.1	0.93 ± 0.01	399 ± 50	0.07 ± 0.006	48
**LQT3**											
G9V	3.5 ± 0.2	−33.4 ± 0.6	6	5.2 ± 0.2	−71.3 ± 0.9	9	3.9 ± 0.3	0.93 ± 0.02	339 ± 64	0.07 ± 0.012	12
R18W	3.6 ± 0.2	−33.8 ± 1.0	6	4.9 ± 0.1	−72.1 ± 1.2	5	4.4 ± 0.4	0.94 ± 0.01	281 ± 48	0.06 ± 0.008	13
V125L	3.8 ± 0.2	−32.9 ± 0.6	25	5.2 ± 0.2	−74.5 ± 1.9	8	3.9 ± 0.3	0.93 ± 0.04	283 ± 64	0.07 ± 0.011	9
**BrS**											
R18Q	3.8 ± 0.1	−32.3 ± 1.6	12	5.5 ± 0.1	−70.6 ± 1.1	11	3.4 ± 0.3	0.93 ± 0.02	236 ± 53	0.07 ± 0.013	11
R27H	3.6 ± 0.1	−32.5 ± 0.9	26	5.5 ± 0.2	−72.2 ± 1.1	6	3.8 ± 0.6	0.91 ± 0.05	505 ± 182	0.09 ± 0.018	5
G35S	3.7 ± 0.2	−33.8 ± 0.8	10	5.2 ± 0.1	−73.3 ± 1.0	9	4.2 ± 0.2	0.92 ± 0.01	452 ± 97	0.08 ± 0.013	14
V95I	3.8 ± 0.3	−31.6 ± 2.4	6	5.1 ± 0.1	−71.6 ± 1.1	11	3.3 ± 0.1	0.90 ± 0.01	134 ± 35	0.10 ± 0.009	5
R104Q	3.9 ± 0.2	−34.0 ± 0.8	7	5.6 ± 0.3	−73.8 ± 0.5^*^	6	4.6 ± 0.1^*^	0.92 ± 0.02	344 ± 125	0.08 ± 0.012	12
K126E	3.7 ± 0.1	−32.9 ± 0.8	24	4.9 ± 0.1	−70.0 ± 0.8	9	3.1 ± 0.2	0.91 ± 0.04	105 ± 32	0.09 ± 0.009	6

* * indicates p < 0.05 vs. hNa_v_1.5*.

In conclusion, G9V channels were indistinguishable from wild-type hNa_v_1.5. R18W showed a decelerated current decay, similarly as seen in some other LQT3 mutant channels (see section Discussion). V125L was characterized by the most severe defects (slower inactivation, increased window current and faster recovery from inactivation). Moreover, our data show that the gain-of-function defects in V125L, typically seen in LQT3 mutant channels, became manifest only in the mammalian expression system.

### Properties of BrS mutant channels (R18Q, R27H, G35S, V95I, R104Q, K126E)

**Figure 4:**
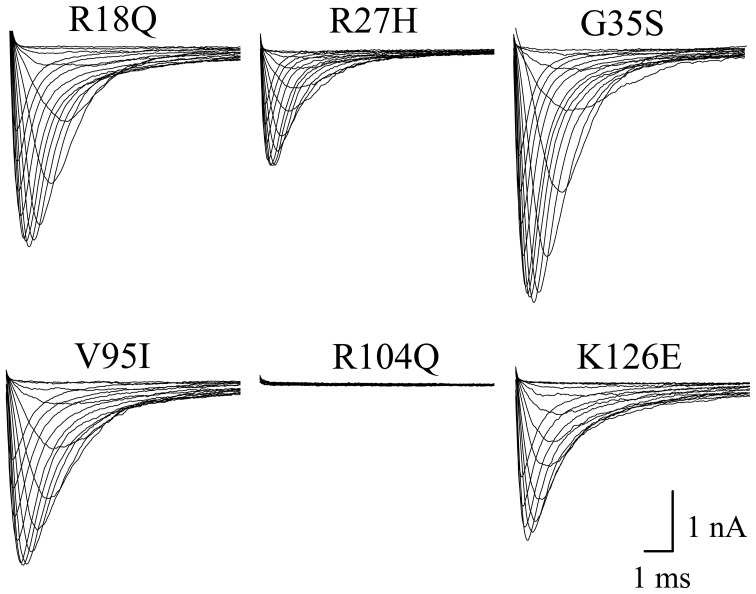
**Whole-cell Na^+^ currents upon expression in HEK293 of hNa_v_1.5 mutant channels associated with BrS.** Currents were elicited from −80 mV to various test pulses in 5 or 10 mV increments at a pulsing frequency of 1.0 Hz (holding potential −120 mV). For average peak current densities see Table 1. Expression of R104Q did not result in functional channels in the mammalian expression system.

Expression of a great number of mutated Na^+^ channel variants, associated with BrS, results either in a significant peak current reduction or even in non-functional channels. Surprisingly, when expressing the six N-terminally mutated variants in HEK293 cells, we did not observe a current reduction in five of them (Table 1, Figure 4). R18Q, R27H, G35S, V95I, and K126E generated whole-cell currents that were comparable to those observed for hNa_v_1.5. Expression of R104Q did not result in functional channels in HEK293 cells (Figure 4). Interestingly, when injecting *Xenopus* oocytes with cRNA for this mutant variant, typical Na^+^ inward currents were observed, but peak currents were reduced to 29% compared to hNa_v_1.5 (Table 1).

**Figure 5:**
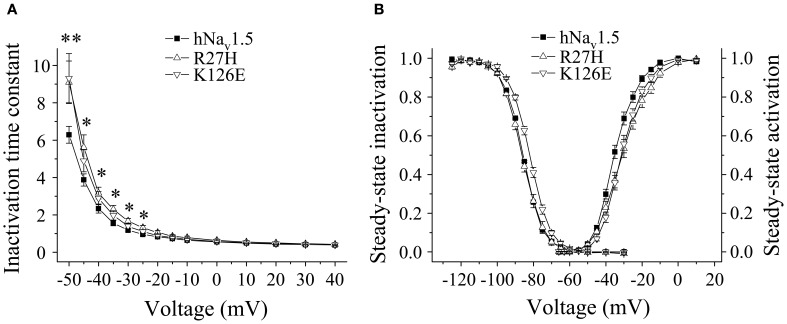
**Electrophysiological properties in HEK293 cells of mutant hNa_v_1.5 channels associated with BrS. (A)** Inactivation time constants τ_*h*_ (ms) as function of voltage. K126E channels inactivated more slowly at −50 mV (^**^), R27H inactivated more slowly from −50 to −25 mV (^*^), when compared to hNa_v_1.5. R18Q, G35S, and V95I channels were indistinguishable from hNa_v_1.5 (data not shown). **(B)** Steady-state activation and steady-state inactivation curves. Mid-activation potentials (*V*_*m*_) were significantly shifted towards depolarized potentials in both R27H and K126E. Mid-inactivation potential *V*_*h*_ was shifted only in K126E. The figure was drawn using the mean of 4 representative measurements for each. *V*_*m*_ and *V*_*h*_ of the other mutant channels, R18Q, G35S and V95I, were indistinguishable from the respective hNa_v_1.5 values. For data and statistics see Table 2.

In HEK293 cells, two out of the five functional mutant channels were characterized by a positive shift of the steady-state activation relationship. In R27H and K126E, the mid-activation potential *V*_*m*_ was shifted by 4.0 mV and 2.8 mV, respectively, and in R27H, the slope was significantly increased (Table 2, Figure 5). This shift in R27H and K126E was accompanied by a respectively slower channel inactivation at less depolarized membrane potentials (Figure 5A). Steady-state activation and inactivation time constants in R18Q, G35S and V95I were unchanged compared to hNa_v_1.5 (Table 2). Analyzing steady-state inactivation in the mammalian expression system, we observed only an increased availability in K126E: The corresponding mid-inactivation potential *V*_*h*_ was by 3.4 mV more positive compared to hNa_v_1.5 (Table 2, Figure 5B). Time constants for recovery from inactivation were not significantly altered in the BrS mutant channels. We only noticed a decreased amplitude of the fast recovery time constant and an increased amplitude of the slow recovery time constant in R27H.

When analyzing our oocyte recordings with the BrS mutant channels, we were very surprised that the electrophysiological parameters for steady-state activation, steady-state inactivation, and recovery from inactivation were statistically indistinguishable from those seen in hNa_v_1.5 (Table 3). None of the loss-of-function defects, observed in R27H and K126E channels in HEK293 cells, were observed in the oocyte expression system.

**Figure 6:**
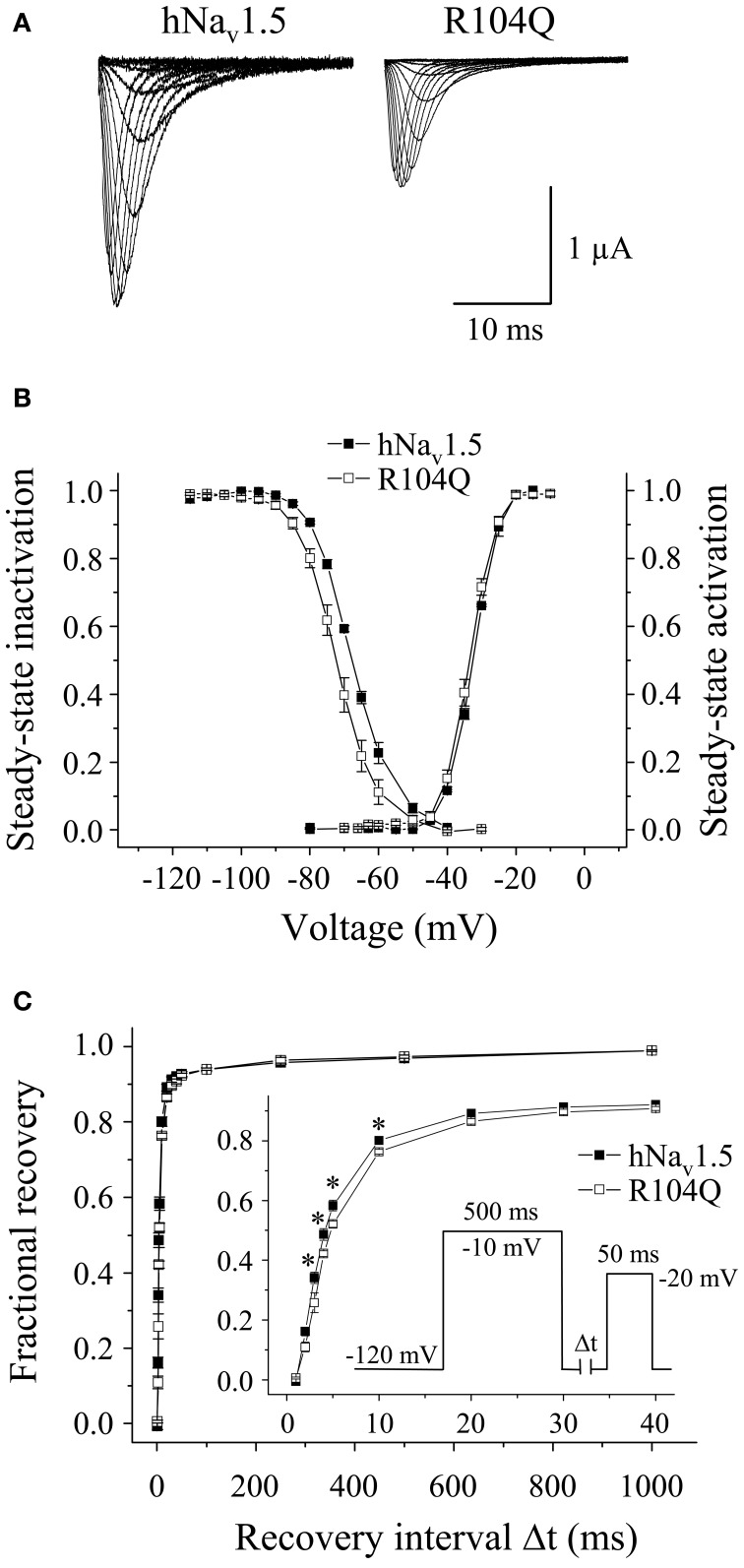
**Electrophysiological properties of R104Q in *Xenopus* oocytes. (A)** Whole-cell Na^+^ currents. Peak current amplitudes were significantly reduced in R104Q (see Table 1). Currents were elicited from −80 mV to various test pulses in 5 or 10 mV increments at a pulsing frequency of 1.0 Hz (holding potential −120 mV). **(B)** Steady-state activation and inactivation curves. In R104Q, steady-state availability was significantly reduced. **(C)** Recovery from inactivation was slower in R104Q (^*^ indicates *p* < 0.05 vs. hNa_v_1.5). For individual values see Table 3.

Functional expression of R104Q in *Xenopus* oocytes allowed us to determine the electrophysiological properties of these mutant channels (Table 3). In addition to reduced peak current densities, we observed a significant reduction in channel availability (Figure 6). The mid-inactivation potential was shifted by 2.6 mV into the hyperpolarized direction (see *V*_*h*_ values in Table 3). At the same time, recovery from inactivation was decelerated in R104Q (Table 3, Figure 6C). Steady-state activation and inactivation time constants remained unchanged when compared to the corresponding hNa_v_1.5 data.

In conclusion, expression of R18Q, G35S, and V95I in HEK293 and *Xenopus* oocytes did not reveal any of the loss-of-function features typically observed in BrS mutant channels. In HEK293 cells, R27H and K126E were characterized by positively shifted steady-state activation curves, a result that is in agreement with a BrS phenotype (Clancy and Kass, 2002). R104Q was not functional in HEK293 cells, whereas in *Xenopus* oocytes we found functional expression but the current amplitude was significantly diminished and the recovery from inactivation was slowed. So it seems that, similarly to the LQT3 mutants, channel defects are more pronounced in the mammalian than in the oocyte expression system.

## Discussion

Our study on the nine N-terminally mutated cardiac Na^+^ channels revealed three main results: First, three mutations produced gain-of-function or loss-of-function defects that are most likely associated with LQT3 (V125L) or BrS (R104Q, R27H), respectively. Second, most of the heterologously expressed channels were either indistinguishable from wild-type hNa_v_1.5 or characterized by only marginally altered electrophysiological properties. And third, in order to detect alterations of the electrophysiological properties in mutant channels mammalian cells were superior to *Xenopus* oocytes.

### Genotype-phenotype correlations for LQT3 mutants

Among the three LQT3 mutant channels investigated, the most pronounced gain-of-function features were noticed in V125L. Despite the absence of an increased persistent current fraction, three other channel defects were observed (Figure 3). These defects may synergistically prolong the cardiac action potential and explain QTc interval prolongation in the patients (Tester et al., 2005). In R18W we found only a slowing of open-state inactivation at less depolarized membrane potentials. It is doubtful that such a rather mild effect is sufficient to cause LQT3. A similar genotype-phenotype disassociation was previously reported in case of P1332L, a mutation that produced only mild channel defects, similarly as R18W (Ruan et al., 2007). P1332L was found in two independent studies and all index patients were kids with QTc values of up to 760 ms (Kehl et al., 2004). At the same time, other mutant channels characterized by a slower current decay also produced a significant persistent current fraction, like Y1795C channels (Rivolta et al., 2001), but patients did not present such extremely prolonged QT intervals. It is further noteworthy that R18W was identified in another study on BrS as a rare control, but not as a mutation causing LQT3 (Kapplinger et al., 2010). Together these observations strongly suggest that additional yet unknown mechanisms contribute to the ECG alteration in R18W and P1332L carriers. G9V mutant channels were even indistinguishable from wild-type hNa_v_1.5. This was the most surprising result, because glycine is highly conserved at this position (Figure 1). One explanation for this obvious discrepancy between our results and clinical data is that the 69 years old patient also presented a homozygous *KCNH2* polymorphism (T897 instead of K897) (Millat et al., 2006). Future studies may answer the question whether or not this rare polymorphism is capable of modulating the cardiac action potential in G9V mutant carriers.

### Genotype-phenotype correlations for BrS mutants

Among the six BrS mutant channels investigated in this study, the most pronounced loss-of-function features were seen in R104Q. Expression in HEK293 cells did not result in functional channels suggesting *SCN5A* haploinsufficiency in R104Q carriers. Similar results were obtained in a recent study on R104W (Clatot et al., 2012). It is interesting to note that even successful expression in *Xenopus* oocytes resulted in significant channel loss-of-function (Figure 6). These data together suggest that position 104 is crucial for Na^+^ channel expression and kinetics. The molecular mechanism leading to loss-of-function in R104Q is still unknown. The lack of a Na^+^ current in R104Q-transfected cells could be due to incorrect folding in the endoplasmic reticulum, impaired post-translational modification, disturbed trafficking, enhanced degradation, or defective gating and permeation in otherwise correctly targeted channels. In R27H and K126E channels, we observed a positive shift of the steady-state activation curve, similarly as seen in other BrS mutants like A735V (Vatta et al., 2002b). Such a shift is expected to decelerate upstroke velocity and cardiac conduction, and thus may explain the observed ECG alterations (Clancy and Kass, 2002; Priori et al., 2002). In K126E, this loss-of-function feature was accompanied by an increased steady-state availability, and thus by a potential gain-of-function feature, suggesting additional yet unknown factors that contribute to BrS in K126E carriers. Nevertheless, we think that the K126E is indeed the primary cause of the disease. First, K126 is a conserved residue and the mutation caused an exchange of a positive charge by a negative amino acid. Second, K126E was detected in two unrelated patients (Vatta et al., 2002a; Kapplinger et al., 2010). And third, a concomitant shift of both steady-state activation and inactivation curves was also reported for other BrS mutant channels, like N406S (Itoh et al., 2005) and delK1479 (Zhang et al., 2007).

In R18Q, G35S, and V95I we could not detect any functional defect (Tables 2, 3), and the pathogenic mechanism leading to BrS remains to be investigated. At least V95 is highly conserved, and it is reasonable to assume that the mutation at this position is correlated to the observed clinical phenotype (Liang et al., 2006). G35 is only conserved among the *SCN5A* orthologs, but not within the Na_v_1 subfamily (Figure 1), which may suggest that G35S is a variant rather than a mutation. However, G35S was not found in 100 controls and, paradoxically, the clinical features of the respective patient were more severe than those seen in case of R104Q carriers (Levy-Nissenbaum et al., 2001).

### Possible reasons for the observed genotype-phenotype disassociations

When analyzing the N-terminally mutated cardiac Na^+^ channels, we noticed dramatic differences between both expression systems. Channel defects were mainly seen in the mammalian system, but not in frog oocytes. Such expression system differences have been occasionally reported (Baroudi et al., 2000). However, it seems that this is rather the exception than the rule, and despite technical limitations of the oocyte system, like variations in oocyte quality and clamp properties, similar data have been observed for most of the hNa_v_1.5 mutant channels. For example, an increased persistent current in ΔKPQ channels can be easily detected in both *Xenopus* oocytes and HEK293 cells (see Table 1). Moreover, in a previous study on nine SSS-related mutant channels, loss-of-function features were similarly detected in both expression systems (Gui et al., 2010). In some cases, even more severe loss-of-function properties were associated with the oocyte system. Interestingly, none of the SSS-related mutations localized to the hNa_v_1.5 N-terminus.

We suggest that the expression system differences in the present study point to important cellular mechanisms or factors in cardiomyocytes that specifically control or modulate the Na^+^ channel N-terminus. It has been shown that the cytoplasmic N-terminal domain of Na_v_1.6 is required for intracellular transport to the plasma membrane, a process that is facilitated by microtubule-associated protein Map1b (Sharkey et al., 2009; O'Brien et al., 2012). *Xenopus* oocytes may not be equipped with respective protein quality control mechanisms, in contrast to HEK293 cells that may accommodate some of those molecular components. To provide evidence for this hypothesis, the N-terminally mutated variants have to be expressed in cardiomyocytes, similarly as done for D1275N channels by Watanabe and co-workers recently (Watanabe et al., 2011). The authors found near-normal currents upon heterologous expression of D1275N, but striking *in vivo* effects in mice carrying one D1275N allele. Similarly, E1053K channels were efficiently targeted to the plasma membrane in HEK293 cells and produced robust currents. The ankyrin-binding motif abolished by this mutation was not required for a successful expression in this cellular system. In cardiomyocytes, however, mutant channels were nearly absent from T-tubules and intercalated discs, and expression at the cell surface was strongly reduced (Mohler et al., 2004).

Other pathogenic mechanisms in monogenetic ion channel diseases, apart from defective trafficking or ankyrin binding, could be an acidic intracellular pH (Wang et al., 2007), increased temperature (Keller et al., 2005), or impaired channel expression from the wild-type and/or the mutated allele (Shang and Dudley, 2005; Leoni et al., 2010; Atack et al., 2011). Moreover, we and others have demonstrated that the cellular splicing machinery is a player affecting genotype-phenotype correlations in cardiac diseases (Shang et al., 2007; Walzik et al., 2011; Murphy et al., 2012). It is possible that some mutant channels investigated in this study show normal electrophysiological features in the background of the wild-type sequence, but altered properties upon an alternative splicing event, similarly as shown previously for T1620K (Walzik et al., 2011). Genetic testing for the diagnosis of *SCN5A* channelopathies does not include analysis of the patients' mRNA, and consequently, possible splicing alterations can not be detected. It is intriguing to speculate that an abnormal splicing could be caused by a pathological alteration of the splicing machinery itself, leading to an excision of important *SCN5A* exons, to the alternative usage of neonatal exon 6a instead of adult exon 6b, or even to an alternative exon usage in another cardiac ion channel. This could result in ion channel defects also in the absence of any ion channel mutation, and thus explain why nearly 80% of all BrS patients are *SCN5A*-negative cases. We think that this is a fascinating idea worth to be tested in the future.

### Conflict of interest statement

The authors declare that the research was conducted in the absence of any commercial or financial relationships that could be construed as a potential conflict of interest.
